# Using Electronic Health Record to Identify Caregivers of People With Dementia in Hospital Settings

**DOI:** 10.1093/geroni/igaf122.038

**Published:** 2025-12-31

**Authors:** Te-Lien Ku, Kayla Dillon, Beth Fields

**Affiliations:** University of Wisconsin-Madison, Madison, Wisconsin, United States; University of Wisconsin–Madison, Madison, Wisconsin, United States; University of Wisconsin–Madison, Madison, Wisconsin, United States

## Abstract

Family and friend caregivers play a crucial role in supporting people living with dementia (PLWD) during hospitalization and post-discharge. Accurate identification and documentation of these caregivers are essential for effective care planning and decision-making. However, identifying caregivers in hospital settings can be challenging, and how these processes involved are less understood in the literature. Using a qualitative description approach, we examined electronic health record (EHR)-based practices during a 10-month hospital-based clinical trial to better understand the challenges and potential solutions in caregiver identification and documentation. Out of the 1,698 screened EHR charts of hospitalized PLWD, only 111 identified a caregiver. Key challenges in identifying caregivers through the EHR included the lack of structured fields in the record for documenting caregivers within the patient’s care network beyond emergency contacts, as well as accompanying details (e.g., relationship to the patient, distance caregiving, power of attorney status, level of care involvement, and contact priority among multiple caregivers). Additionally, inconsistencies in documentation practices in the EHR across different hospital units further complicate efforts to standardize caregiver identification. To address these challenges, we propose several solutions to enhance caregiver identification and documentation for practice and research. These include integrating structured EHR fields that go beyond emergency contacts and embedding prompts and automated alerts to facilitate early and systematic caregiver recognition for clinicians. Refining documentation workflows and strengthening caregiver identification will ensure the most appropriate caregivers receive the necessary information and resources as needed.

